# Medical education engagement during the COVID-19 era – A student parents perspective

**DOI:** 10.1080/10872981.2020.1788799

**Published:** 2020-07-01

**Authors:** Lola Arowoshola

**Affiliations:** Institute of Medical and Biomedical Education, St. George’s, University of London, London, UK

**Keywords:** Medical education, student parents, online teaching; home-schooling, education engagement

## Abstract

The COVID-19 pandemic has affected the delivery of medical education and has limited the ability of student parents to fully engage with their studies. Student parents have been faced with additional challenges such as increased childcare roles and home-schooling responsibilities, splitting their focus. Identifying the issues student parents face and adopting workable solutions at all levels, will ensure the best outcomes for these students and better preparedness for the future.

The Coronavirus (COVID-19) pandemic has had far reaching implications worldwide. Society and socialising have been significantly changed with the imposition of infection control measures such as social distancing, the use of facemasks and the enforcement of nationwide lockdowns and travel restrictions. Work-from-home is now the new normal for the majority of the population in lockdown. Educational institutions ranging from nurseries and primary schools all the way to up further education establishments have been closed except to children of key workers [1]. This has led to parents providing round the clock childcare while still working or studying full time. Parents have also become impromptu teachers as home-schooling has been adopted. The change in the educational landscape is likely to have significant and unforeseen consequences for years to come [2].

Medical education has not been spared in these COVID-19 times. In the UK, just prior to the official government lockdown on 23rd March 2020, some medical schools began cancelling clinical placements and face-to-face teaching [3]. At St George’s University of London, the teaching and administrative staff developed online teaching content to be delivered via platforms such as Big Blue Button, Zoom and Microsoft Teams. The use of technology to provide online teaching has been shown to achieve high levels of student engagement [4]. The content covered for missed placements, small group tutorials, clinical skills sessions, anatomy sessions as well as lectures.

The availability and accessibility of such online resources is however not synonymous with student engagement for some subsets of the student cohort. Medical students with parental responsibilities fall into this group. Under normal circumstances, student parents face many challenges that affect their educational experience and their ability to engage [5]. Specifically, the requirement for care giving and home-schooling during the lockdown has affected my ability to engage fully with my studies. Home-schooling can be a very involved process especially for younger children. Children in reception/Year 1 are just at the start of their education, when it is essential they build the basic foundations for a life of learning. They require consistent teaching and interaction due to the potential for decline in school performance during lockdown [2]. For me, this raised the dilemma where I was not able to focus fully on teaching my children properly as I still had to give attention to my medical education. As such, I struggled to do justice to either. Online teaching sessions running for several hours would lead to interruptions and reduced focus due to attending to the caring needs of my children. To compound matters further, the need to prepare for and sit an online exam from home with all the associated stresses and distractions made a hard situation even worse.

The medical faculty provided different avenues to help students with caring needs. These ranged from the extreme of taking an Interruption of Studies to providing extra time to submit any coursework and ensuring all online teaching was recorded, allowing students the option to gain from the session at a more convenient time.

During the lockdown period, the Department of Health named medical students as essential workers [6]. This title has given student parents the opportunity to send their children to school on a full time basis, giving some much needed assistance. In theory, this solution would seem to solve the childcare problem facing student parents. However, access has been mixed as not all nurseries or schools have been able to remain open for key workers children. Some schools have required that in two parent households, both parents must be key workers to be allowed to attend. Schools are only running standard hours of 9am to 3pm, which do not align with full time attendance and placement hours. This government level solution has unfortunately not fully addressed the needs of all essential workers, which extends to student parents. As lockdown eases in England, the working plan for schools to reopen fully in September, may not be realised [7]. We also have to be wary as the potential for isolation measures to be continued as far as 2022 is a possibility [8]. There is a lot of uncertainty looming. This will invariably continue to impact on student parents ability to engage fully with their course and affect their ability to attain high levels of academic achievement.

In times of uncertainty and crisis, challenges can be quickly identified in isolation and the solutions developed and implemented can have limited effect. Although the situation around COVID-19 is novel and unforeseen, the approach taken to appropriately manage a crisis is essential. What COVID-19 has taught us as societies is the need for flexibility and adaptability. This extends from the government, to the medical institutions and to us students as well. It is important that we take the time to identify the issues appropriately. By taking note of the success stories such as online teaching in medical education, we have the opportunity to improve and develop them further. We must also learn from the shortcomings of the solutions adopted, especially those at the government level so we are better prepared for next time. The health service requires medical students to progress and graduate, so the onus is on all parties to ensure that no student is left behind.
